# Supplementary material for: The Systems Biology Research Tool: evolvable open-source software
Source: BMC Syst Biol. 2008 Jun 29;2:55. doi: 10.1186/1752-0509-2-55 (PMC2446383; doi:10.1186/1752-0509-2-55)
Supplement: Additional file 1 — SBRT Archive. An archive of the current version of the Systems Biology Research Tool. [file 1752-0509-2-55-S1.zip › sbrt-1.4.0/doc/developers_guide/api/sbrt/shell/io/MultiOutputFiles.html]

MultiOutputFiles


|  |  |  |
| --- | --- | --- |
| **Overview** \| **Package** \| **Class** \| **Use** \| **Tree** \| **Deprecated** \| **Index** \| **Help** | |  |
| **PREV CLASS**   **NEXT CLASS** | **FRAMES**    **NO FRAMES**     **All Classes** |  |
| SUMMARY: NESTED \| FIELD \| CONSTR \| METHOD | DETAIL: FIELD \| CONSTR \| METHOD |  |


---


## sbrt.shell.io Class MultiOutputFiles<F extends OutputFile<R>,R>

```
java.lang.Object
  sbrt.shell.io.MultiOutputFiles<F,R>
```

**Type Parameters:**: `F` - the file type.: `R` - the result type.

**All Implemented Interfaces:**: ResultsManager<R>

---

``` public class MultiOutputFiles<F extends OutputFile<R>,R> extends java.lang.Object implements ResultsManager<R> ```

This class is used to create multiple output files.

**Author:**
:   This class was written and documented by
    Jeremiah Wright while in the Wagner lab.

---

**Constructor Summary**

`MultiOutputFiles(java.util.Iterator<F> fileSource)`
  
          Constructs a new multiple output files results manager.


| **Method Summary** | |
| --- | --- |
| `<S extends R> void` | `addData(S results)`             Adds the provided results to the current file. |
| `void` | `breakPoint()`             Closes the current file if one exists. |
| `void` | `close()`             Closes this results manager so that no further results can be added. |
| `java.lang.String` | `getName()`             Returns the name of the current file. |
| `boolean` | `isClosed()`             Indicates if this results manager has already been closed. |

**Methods inherited from class java.lang.Object**

`clone, equals, finalize, getClass, hashCode, notify, notifyAll, toString, wait, wait, wait`

**Constructor Detail**

### MultiOutputFiles

```
public MultiOutputFiles(java.util.Iterator<F> fileSource)
```

:   Constructs a new multiple output files results manager.

    **Parameters:**: `fileSource` - a source of output files.


**Method Detail**

### close

```
public void close()
```

:   Closes this results manager so that no further
    results can be added. If already closed, then
    invoking this method has no effect.

    :   **Specified by:**: `close` in interface `ResultsManager<R>`

---


### isClosed

```
public boolean isClosed()
```

:   Indicates if this results manager has already
    been closed.

    :   **Specified by:**: `isClosed` in interface `ResultsManager<R>`
    :   **Returns:**: `true` if results can still be added to this manager; `false` otherwise. **See Also:**: `close()`

---


### addData

```
public <S extends R> void addData(S results)
```

:   Adds the provided results to the current file. If a current
    file does not already exist, one is created and the
    provided results are added to it.

    :   **Specified by:**: `addData` in interface `ResultsManager<R>`
    :   **Type Parameters:**: `S` - a type extending the primary results type. **Parameters:**: `results` - the results to add to the current file.

---


### getName

```
public java.lang.String getName()
```

:   Returns the name of the current file.

    :   **Specified by:**: `getName` in interface `ResultsManager<R>`
    :   **Returns:**: the name of the current file, or `null` if one does not exist.

---


### breakPoint

```
public void breakPoint()
```

:   Closes the current file if one exists.

    :   **Specified by:**: `breakPoint` in interface `ResultsManager<R>`


---


|  |  |  |
| --- | --- | --- |
| **Overview** \| **Package** \| **Class** \| **Use** \| **Tree** \| **Deprecated** \| **Index** \| **Help** | |  |
| **PREV CLASS**   **NEXT CLASS** | **FRAMES**    **NO FRAMES**     **All Classes** |  |
| SUMMARY: NESTED \| FIELD \| CONSTR \| METHOD | DETAIL: FIELD \| CONSTR \| METHOD |  |


---
